# Pre-Grazing Herbage Mass Affects Grazing Behavior, Herbage Disappearance, and the Residual Nutritive Value of a Pasture during the First Grazing Session

**DOI:** 10.3390/ani10020212

**Published:** 2020-01-28

**Authors:** Luis F. Piña, Oscar A. Balocchi, Juan Pablo Keim, Rubén G. Pulido, Felipe Rosas

**Affiliations:** 1 Escuela de Graduados, Facultad de Ciencias Agrarias, Universidad Austral de Chile, P.O. Box 567, Valdivia 5090000, Chile; luispina@uchile.cl (L.F.P.); felipe_rosas77@hotmail.com (F.R.); 2 Departamento de Producción Animal, Facultad de Ciencias Agronómicas, Universidad de Chile, Santiago 8820808, Chile; 3 Instituto de Producción Animal, Facultad de Ciencias Agrarias, Universidad Austral de Chile, P.O. Box 567, Valdivia 5090000, Chile; juan.keim@uach.cl; 4 Instituto de Ciencia Animal, Facultad de Ciencias Veterinarias, Universidad Austral de Chile, P.O. Box 567, Valdivia 5090000, Chile; rpulido@uach.cl

**Keywords:** herbage mass, nutrient selection, ingestive behavior, dry matter intake, herbage disappearance

## Abstract

**Simple Summary:**

The progressive defoliation carried out by dairy cows during the grazing-down process affects the characteristics of the pasture, the dry matter intake and the productive performance of the animals, particularly during the first hours of grazing. This is especially relevant when increasing the efficiency in pasture-based dairy systems. Two pre-grazing herbage masses (high and medium herbage masses) were used to evaluate the process of defoliation carried out by dairy cows during the first hours after the beginning of grazing and its effects on the pasture and animals. The pre-grazing herbage mass affected the ingestive behavior of dairy cows, which influenced the productive performance, and the morphological characteristics and nutritive value of the pasture. It is concluded that pre-grazing herbage mass affects the grazing process carried out by dairy cows during the first hours after the allocation of a new grazing area, modifying the eating pattern of the dairy cows. The results of our study allow highlighting the importance of grazing management in pasture-based dairy systems.

**Abstract:**

During the first hours after the allocation of a grazing strip (first grazing session, GS), dairy cows eat most of the daily dry matter (DM) available. There are few studies that analyze how the grazing-down process changes the characteristics of the pasture during the first GS. The objective of this study was to evaluate the effect of two pre-grazing herbage masses (HM; medium herbage mass (MHM) and high herbage mass (HHM) on the DM disappearance, grazing behavior of dairy cows, and the residual nutritive value of a pasture during the first GS. Two groups of twelve dairy cows were used to evaluate the grazing-down process, during a period of 62 days. The pre-grazing HM modified the bite rate, bite mass, and dry matter intake during the first GS. The pre-grazing HM affected the process of herbage disappearance of the pasture, especially during the first 60 min of the GS. The nutrient selection differential for acid detergent fiber was greater for HHM compared with MHM (0.93 vs. 0.86). In conclusion, pre-grazing HM affects the structural characteristics and the residual nutritive value of the pasture. The grazing process in the first GS was modified by the HM, affecting the defoliation and the DM disappearance rate of the pasture.

## 1. Introduction

Herbage mass (HM) is one of the key factors that influence dry matter intake (DMI) in grazing dairy cows [1,2]. In homogeneous pastures it is expected that HM is related with the nutritive value of the forage offered and therefore DMI rate. However, during the grazing process, changes from the initial state of the pasture occur that generate high heterogeneity in the forage available to animals [3], in terms of both the nutritive value and the structural characteristics that influence the foraging behavior of dairy cows.

In grazing systems based on strip allocations, particularly with an herbage allowance (HA) equivalent to the potential DMI of animals, dairy cows eat most of the daily dry matter (DM) available in the first grazing session (GS). In strip-grazing systems, the first GS begins immediately after the allocation of the grazing strip, and the most significant changes in the structure of the pasture and in the grazing behavior of the animals, occur during the first 240 min of grazing [4]. Thus, the study of the eating patterns used by dairy cows during the grazing-down process in this first GS becomes especially relevant to maximize the grazing efficiency and the productive performance of dairy cows.

Pre-grazing HM directly influences the leaf:stem ratio existing in the different strata of the pasture and, therefore this variable influences the bite mass of dairy cows [5]. However, due to their location in the canopy, leaves are the first structures selected and consumed by grazing dairy cows, particularly those located in the upper strata of the pasture, and thus the grazing-down process in the pasture will influence its nutritional value, the ease of harvest and the DMI of the cows [6].

Even though it is well defined how HM affects DMI, grazing behavior and milk responses of dairy cows [1,7,8], there are few studies that analyze in detail how the grazing behavior of dairy cows changes the characteristics of the pasture during the first GS [4,9], particularly how progressive defoliation affects the DM disappearance and causes structural modifications in the pasture and its nutritional value throughout the GS, which may in part explain the changes in grazing behavior, selection for nutrients, DMI and productive responses of grazing dairy cows.

Thus, the objective of this study was to evaluate the effect of pre-grazing HM on the DM disappearance, grazing behavior of dairy cows, and the residual nutritive value of a pasture during the first grazing session.

## 2. Materials and Methods

### 2.1. Experimental Location

The experiment was conducted at the Austral Agricultural Research Station of Austral University of Chile (39°47′ S, 73°13′ W, Valdivia, Chile) during 62 days in spring of 2017 (from October 6 to December 13). An average air temperature of 12.6 °C was recorded during the 62-days trial period. In the same period, accumulated rainfall of 178.9 mm was recorded. The amount of accumulated rainfall was equivalent to 81.8% of the 5-year average (218.7 mm) for the period of the study. The air temperature was 0.5 °C lower than the average of the last 5 years (13.1 °C) between the months of October and December. The dominant species in the pasture were perennial ryegrass (*Lolium perenne* L.; 86%) and white clover (*Trifolium repens* L.; 6%). The pasture was established two years earlier and subjected to strip-grazing management. The test site soil was Andisol (Duric-Typic Hapludand), characterized by high organic matter content, low pH, and high phosphorus retention [10].

### 2.2. Treatments, Animals, and Experimental Design

All procedures in this experiment were approved by the Animal Welfare Committee of the Universidad Austral de Chile (grant number 255/2016).

A completely randomized design was used to evaluate two contrasting pre-grazing HMs, considering all herbage above ground level (>0 cm): high herbage mass (3400 kg DM/ha, HHM) and medium herbage mass (2800 kg DM/ha, MHM).

Two groups of 12 Holstein-Friesian cows (n = 12) were balanced according to the number of lactations (2.88 ± 1.7), days in milk (50.3 ± 11.8 days), milk production (31.6 ± 8 kg/cow/day), and body weight (501.2 ± 74 kg), and were assigned to the grazing treatments. The experiment lasted 62 days including a 7-day adaptation period and a 55-day measurement period. A 5.4 ha paddock was used, which was divided into two areas to establish the pre-grazing HM treatments. Forty days before the start of the experiment, the pasture was grazed, leaving 4-cm stubble. The area meant for the MHM treatment was grazed again 15 days before the start of the experiment to produce the difference in pre-grazing HM associated with the HHM treatment. The grazing strip was considered as the experimental unit to analyze the variables related to the pasture.

### 2.3. Animal Handling and Grazing Management

Rotational grazing was used with two grazing strips per day (08:00 and 17:30) after each milking. A pasture allowance of 25 kg DM/cow/day (>0 cm), divided equally for each strip, was assigned. The grazing area for each treatment was calculated based on the daily measurements of pre-grazing HM (>0 cm) and the pasture allowance. The pasture strips were divided with electric fences in front of and behind each strip to prevent the cows from grazing over a previously grazed strip. The cows were milked twice a day (07:00 and 16:00) and were supplemented with 5.2 kg DM/cow/day of concentrate in individual feeders during a.m. and p.m. milkings.

### 2.4. Pasture Measurements

Pre- and post-grazing compressed height (cm) were measured in each pasture strip using a rising plate pasture meter (Jenquip, Feilding, New Zealand) to estimate HM (kg DM/ha; >0 cm). Fifty measurements of the above variables were collected for each grazing strip following a zigzag pattern. The compressed height data was transformed into kg DM/ha by using a specific equation for spring swards of southern Chile [11]. The equation used is as follows:

(1) Y=100x+400

where *Y* is HM expressed in kg DM/ha, and *x* is averaged compressed height.

Defoliation depth (cm) was estimated by subtracting pre- and post-grazing compressed height. Lamina length (cm) and width (mm) were measured in the second fully developed leaf of 100 randomly selected tillers every 15 days.

Eight pasture “cores” of 78.5 cm^2^ were collected every 15 days, and the tiller density (number of tillers m^−2^) was determined by counting the number of tillers present in the samples.

The morphological composition of the herbage was determined from handfuls of herbage harvested every 15 d. Before the start of the first GS and at the end of this (after 240 min of grazing), fifteen handfuls of herbage were randomly picked at pasture and cut with scissors above 4 cm. Then, the samples were placed in plastic bags and stored at −20 °C until their processing. Each sample was manually separated into four components: Ryegrass leaves, ryegrass pseudostem, dicotyledoneous and dead material, and each component was dried (60 °C for 48 h) to determine morphological composition on a DM basis.

To evaluate the process of DM disappearance from the pasture, the HM was estimated using a rising plate meter, measuring HM prior the grazing event started and then every 30-min during the first 240 min. Herbage mass disappearance rate was calculated by fitting the HM values from each treatment to the exponential model proposed by Ørskov and McDonald [12]:

(2) $$Y=a-b\;\left(1-e^{\left(-c\;x\;time\right)}\right)$$

where *a* represents the initial herbage mass; *b* is the potential HM disappearance; and *c*, is the fractional disappearance rate of the HM.

Parameters of the model were estimated using GenStat 12.1. Instantaneous HM disappearance rates were estimated for 30, 60, 120, 180, and 240 min after the start of the GS as the first derivative of the proposed model.

To evaluate the chemical composition of the pastures, five composite samples were collected per treatment for the pre-grazing period and 60, 120, 180, and 240 min after the start of the GS by harvesting via handpicking strips in the morning and evening [13] to simulate the pasture selection of the cows. Sampling was repeated three times every 15 days, and each sample was analyzed separately. This measurement was made on the same days as the grazing behavior measurements. The samples were stored in plastic bags, weighed in the laboratory (fresh weight), and dried in an oven at 60 °C for 48 h to determine the DM content. Samples were ground using a laboratory mill (Thomas Wiley model 4, Arthur Thomas and Co., Philadelphia, PA, USA), passed through a 1-mm sieve and subsequently analyzed for crude protein (CP), neutral detergent fiber (NDF), acid detergent fiber (ADF), and digestible organic matter in the DM (DOMD) by near-infrared spectroscopy (NIRS, Foss NIR Systems 6500).

The nutrient selection differential was calculated using the procedure described by Wales et al. [14]. The concentration of nutrients selected (*Nutsel*; g/kg DMI) by cows was calculated with the following equation:

(3) $$Nutsel=\frac{\left(HMpre\;\;Nutpre\right)-\left(HMres\;\;Nutres\right)}{HMpre-HMres}$$

where *HMpre* and *HMres* are the HM (kg DM/ha) of the pre-grazing HM and the residual HM after the 240 min of the GS, respectively, and *Nutpre* and *Nutres* are the concentrations of the nutrients (g/kg DM) in the pre-grazing herbage and in the residual HM at the end of the first GS, respectively. The selection differential was calculated by dividing *Nutsel* for *Nutpre*.

### 2.5. Animal Measurements

Milk yield was measured at am and pm milking for each cow using an electronic milk meter (MPC580 DeLaval, Tumba, Sweden). Milk fat and milk protein concentrations were measured individually during two consecutive milkings/week every 15 days, by near-infrared spectrophotometry using a Milkoscan instrument (Foss Electric, HillerØd, Denmark). The body weight (BW) of each cow was recorded automatically once daily after the morning milking. The body condition score (BCS) was estimated every 15 days based on a 1 to 5 scoring system with 0.25 increments [15] by trained operators.

Four trained observers recorded the grazing behavior by observing the cows’ activity over 3 nonconsecutive days every 15 days (four measurement periods). Grazing behavior was defined based on the following animal activities: Grazing (bite procurement, chewing between bites, and/or searching), rumination (standing or lying behavior of the animal), resting (standing or lying behavior of the animal without rumination), and other activities (social interactions, drinking, demonstration of estrus, and others). The activity of individual cows was recorded every 10 min over the course of 240 min after the start of the GS. Grazing behavior was recorded only when animals were in the pasture. The duration of an activity was calculated by summing the duration of the activity across all 10-min intervals. Large numbers painted on the sides of the cows aided identification.

The bite rate (bites/min) was recorded individually every 30 min during the first 240 min after the grazing process began. This measurement was made on the same days as the grazing behavior measurements.

Herbage intake for each time interval of the GS was calculated as the difference between pre-grazing HM and HM at the end of each time interval of the GS [16].

The number of bites per cow during each time interval of the GS was calculated by multiplying the bite rate and eating time of the corresponding time interval. Thus, the bite mass of each time interval was calculated by dividing the herbage intake per time interval for the corresponding number of bites per cow.

### 2.6. Statistical Analyses

The variables related to the pasture characteristics, the parameters of the model of DM disappearance, the instantaneous HM disappearance rate and the nutritive value of the forage were analyzed using a generalized linear model (GenStat 12.1, VNSi, UK). The fixed effects considered were pre-grazing HM (MHM and HHM), and day of measurement. The effect of HM on the nutritive value of the pasture was analyzed at each time point of the first GS (every 60 min), using the model mentioned previously.

Grazing behavior, bite rate, bite mass, herbage intake, and productive performance of the cows were analyzed based on a mixed model (NCSS 2019, LLC, Kaysville, UT, USA). The fixed effects considered were pre-grazing HM (MHM and HHM), and the random effects were the individual cows and day of measurement. The latter effect was treated as a repeated measure, and the variance-covariance structure was selected per variable using the lowest AIC (Akaike information criterion) value as a criterion. Unstructured covariance, autoregressive order one and compound symmetry variance-covariance structures were tested. Grazing and ingestive behavior variables were analyzed at each time interval of 60 min, using the model mentioned above. Thus, the comparison between treatments was realized within time interval. Significance was declared at *p* < 0.05. Model diagnostics included testing for normal distribution of error residuals and homogeneity of variances. All data met the assumptions.

Initially, the factor associated with the time of the day of grazing strip allocation (AM, PM) was included in the analysis. However, this factor was not statistically significant for any variable evaluated in the study, so it was not considered in the final analyses.

## 3. Results

### 3.1. Sward Measurements and Defoliation Characteristics

The effects of pre-grazing HM on sward and defoliation characteristics are shown in Table 1. Pre-grazing HM and pasture height were greater (*p* < 0.05) for HHM compared to MHM (+711.3 kg DM/ha and +3.5 cm, respectively).

Similarly, free leaf lamina length and lamina width were greater (*p* < 0.05) in HHM than in MHM (+5.2 cm and +1.2 mm, respectively. There were no differences between treatments (*p* > 0.05) in terms of post-grazing HM or post-grazing pasture height. The tiller density was higher (+1700 tillers/m^2^; *p* = 0.041) in MHM than in HHM.

The amount of pre-grazing ryegrass leaves was greater in MHM (+239.0 g/kg DM; *p* < 0.001) compared to HHM, whereas the amount of pre-grazing ryegrass pseudostem was greater in HHM (+174.1 g/kg DM; *p* < 0.001) compared to MHM. There were no differences between treatments (*p* > 0.05) in the amount of pre-grazing dicotyledoneous and dead material. There were no differences between treatments (*p* > 0.05) in morphological composition of the sward after the first GS 240 min of grazing.

A trend (*p* = 0.053) in the effect of HM on defoliation depth was observed, being numerically greater in the HHM treatment in relation to MHM treatment (+3 cm). The forage removed per cow was higher (+2.1 kg DM/cow/d; *p* = 0.046) in HHM in relation to HHM, while the area allocated per cow was greater (+19.5 m^2^/cow/d; *p* = 0.019) in the MHM treatment.

### 3.2. Animal Performance, Body Condition Score, and Body Weight

The effect of HM on the cows’ productive performance, BCS and BW are shown in Table 2. Milk production was 1.23 kg/cow/d greater (*p* = 0.021) in the MHM than HHM, whereas milk fat concentration was higher in HHM (42.9 v/s 39.8) than in MHM (*p* < 0.001). No effects of HM (*p* > 0.05) on milk protein concentration, milk fat or protein production, BCS or BW were observed.

### 3.3. Grazing Behavior, Bite Rate, Bite Mass, and Herbage Intake during the First GS

The effects of HM on grazing behavior and DMI during the first GS are shown in Table 3. No effect (*p* > 0.05) of HM on grazing behavior during the first GS was observed. A trend (*p* < 0.1) towards a longer grazing and rumination time in the HHM treatment was observed in relation to MHM in the 120–180 min and 60–120 min time intervals, respectively. Thus, the dairy cows in both treatments expended a similar amount of time for grazing, rumination and idling in each interval of the first GS. The bite rate was higher (*p* < 0.05) in MHM at each moment of measurement during the first GS, with an average of +8.09 bites/min in MHM in relation to HHM. During the first 60 min of the GS, the bite mass was significantly higher (+0.56 g DM; *p* = 0.001) in the HHM compared with MHM. There was no effect (*p* > 0.05) of the HM on bite mass for the rest of the first GS. DMI was higher (+1.08 kg DM/cow; *p* = 0.032) in HHM compared with MHM during the first 60 min of the first GS, whereas there was no effect (*p* > 0.05) of pre-grazing HM for the rest of the GS.

### 3.4. Herbage Disappearance Dynamics

**Figure 1:**
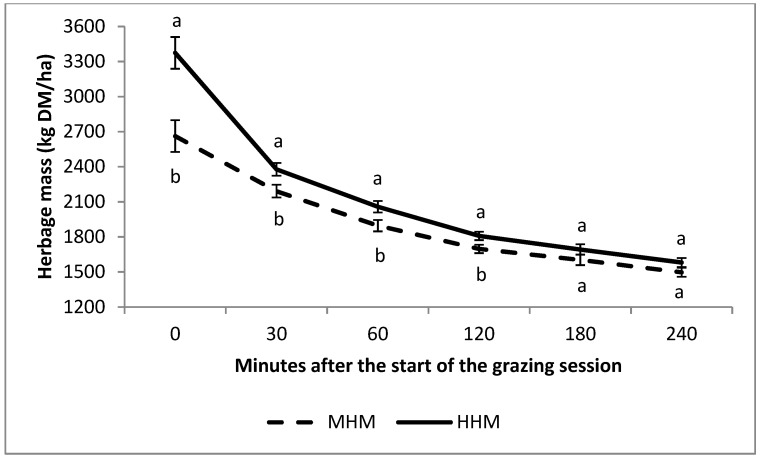
Herbage mass during the grazing-down process in the first grazing session in a medium (MHM) and high (HHM) pre-grazing herbage mass pasture. Different superscript letters at the same time point imply significant differences between treatments (*p* < 0.05).

The process of DM disappearance from the pasture can be observed in Figure 1. From the beginning of the first GS until 30 min after the start of grazing, the herbage disappearance represented 17.7% and 29.5% of the total HM offered in the MHM and HHM, respectively. The values of the parameters *a* (initial HM) and *b* (potential disappearance of DM) of the HM disappearance model (Table 4) differed between treatments (*p* < 0.05), while there was no effect (*p* = 0.510) for *c*. At 30 and 60 min after the GS started, the instantaneous rate of disappearance of DM was higher (*p* < 0.05) in HHM than in MHM, while at 120, 180, and 240 min, there were no significant differences between treatments (*p* > 0.05).

### 3.5. Evolution of Nutrient Concentration of the Pasture throughout the GS and Nutrient Selection Differentials

The effect of HM on the nutrient concentration of the pasture throughout the first GS is reported in Table 5. There were no differences between treatments (*p* > 0.05) in CP concentration at the beginning of the GS and at 120 min after grazing. A trend towards a higher CP content in MHM compared with HHM was observed at 60 and 180 min, (*p* = 0.064 and *p* = 0.057, respectively), whereas CP concentration was significantly higher (*p* = 0.022) for MHM at 240 min.

The ADF content was higher (*p* < 0.05) for HHM compared with MHM during the first 120 min of the GS and a trend was observed (*p* = 0.096) towards a higher ADF content in HHM at 180 min, whereas no difference (*p* > 0.05) between treatments was observed after 240 min of grazing. A trend (*p* = 0.08) towards a higher NDF content in HHM compared with MHM was observed before the GS started. There were no differences (*p* > 0.05) at 60 min after grazing, then NDF content was higher (*P* = 0.02) in HHM compared with MHM at 120 min and there was no HM effect (*p* > 0.05) on NDF concentration at 180 and 240 min. There was no HM effect (*p* > 0.05) on DOMD throughout the GS, except for a trend (*p* = 0.07) towards a greater DOMD for MHM at 120 min.

The nutrient selection differential for ADF was greater (*p* = 0.032) for HHM compared with MHM (Table 6). On the opposite, a trend (*p* = 0.084) towards a greater selection differential for DOMD was observed in MHM compared with HHM. There was no HM effect (*p* > 0.05) on the selection differential for CP and NDF.

## 4. Discussion

### 4.1. Pasture Characteristics, Forage Removal, and Animal Performance

The results of our study show that an increase in HM causes changes in the structure of the pasture, resulting in an increase in the pasture height and size of the plants, which is expressed in a longer length [2] and lamina width. These structural characteristics promote an increase in DMI [17] in the first 60 min of the GS, that was reflected in the greater amount of forage removed in the HHM treatment (Table 1), which is consistent with the results obtained by McEvoy et al. [2] and Wales et al. [14].

In the present study, it was observed that MHM contained lower amounts of pre-grazing ryegrass pseudostems, but higher concentration of pre-grazing ryegrass leaves than HHM. Similar findings have been reported in other studies that analyzed the effect of HM on pasture characteristics and DMI [2,18,19]. The content of pre-grazing ryegrass leaves reported in our study was higher than observed by Muñoz et al. [18], and similar to those reported by Wims et al. [19] and Curran et al. [1], particularly in MHM. This is due to the fact that, in our work and in Wims et al. [19] and Curran et al. [1], the pasture samples were taken above 4 cm, while in Muñoz et al. [18], the samples were taken at ground level. The content of pre-grazing ryegrass pseudostems was higher in HHM in relation to MHM, which according to Tharmaraj et al. [20] would cause a negative effect on the DMI due to the increase in pasture resistance to prehension. According to Curran et al. [1], the negative effect of a high HM on DMI due to the increase in the proportion of stems and the resistance offered to the grazing process by them, would be reduced with a high post-grazing HM which coincides with the results of our study, where the post-grazing HM was greater than 1500 kg DM/ha in both treatments.

These results contrast with that reported by Muñoz et al. [18], who obtained a higher DMI with lower pre-grazing HM evaluated above 3 cm, however the difference in pre-grazing HM among treatments was ca. 3000 kg DM/ha and therefore was affected by differences in the proportion of pseudostems, and therefore different nutritional value of the forage. Thus, the comparison of DMI between experiments should be carried out with caution since the results will depend on the height of the allocation of herbage allowance [2,8,21]. According to Pérez-Prieto et al. [8], when comparing the effect of the HM at the same herbage allowance above ground level, it is observed a positive effect of the HM on DMI, which is consistent with our experiment. On the other hand, when comparing HM at the same herbage allowance above 4 or 5 cm, it was observed a negative effect of HM on DMI [1].

In our experiment, the nutritive value of pre-grazing pasture was relatively similar between treatments, so the structural characteristics of the pasture could become more relevant to the forage removed than the nutritional value of the pasture [21].

Despite the greater amount of pasture removed in the HHM treatment, milk production was higher in the MHM treatment, which is consistent with the results obtained by Muñoz et al. [18] and Curran et al. [1]. The higher milk production in pastures managed with low HM compared to high HM is related to a higher nutritive value of the forage offered [8,18], which was not observed in our study. According to Amaral et al. [22], treatments that promote an increase in defoliation depth, result in a higher proportion of stems and sheaths consumed by dairy cows which makes the forage selection process more difficult. In our study, a tendency towards a greater defoliation depth was observed in the HHM treatment, which could have influenced diet selection by the cows in that treatment. Moreover, we observed that MHM cows had a lower nutrient selection for ADF and tended to a greater selection of DOMD compared with HHM cows, which could explain the greater milk production in the MHM treatment. On the other hand, the increase in milk fat concentration under HHM is in accordance with other studies normally linked to relatively higher fiber concentrations [19].

### 4.2. Grazing Behavior, Herbage Intake, and Herbage Disappearance during the First Grazing Session

During the first GS, grazing behavior was not affected by the HM treatments, with similar grazing, rumination and idling times. The increase in bite rate was the behavioral adaptation of MHM cows trying to maintain a high DMI, whereas in HHM the structure of the pasture (longer lamina length and greater lamina width) allowed a higher bite mass [22,23]. However, it should be noted that the process of DMI during the GS generated changes in the pasture structure in both treatments, which indicates that only during the first 60 min after the start of the GS the bite mass was greater in HHM.

Several studies have indicated that bite mass is the most influential component of the DMI in grazing dairy cows [4,23,24], thus cows offered a HHM presented a higher DMI during the first 60 min of the GS, associated with the greater bite mass registered in this treatment during that time interval [22]. Thereafter, the bite mass was similar between treatments, due to the decrease in pasture height and therefore the available HM, which is consistent with the results of Stakelum and Dillon [25] and Gregorini et al. [4]. Bite rate was higher in MHM throughout the whole GS, which is a behavioral adaptation of cows to the pasture height and available HM to maintain daily DMI [24].

During the first 60 min of the GS, DM disappearance was greater in the HHM treatment than in the MHM treatment, which is consistent with the greater bite mass and amount of pasture removed in this treatment. After 60 min, the process was similar between treatments according to the ingestive behavior of the cows [22]. The ease of the grazing-down process is reduced to the extent that the cows graze lower strata of the pasture, an effect that increases with high HM, which would explain the similarity in DM disappearance between the treatments after the first 60 min of the GS [19,22].

It is important to highlight the meaning of the first GS on ingestive behavior and DMI [26]. The pasture removed after 24 h of grazing (Table 1) is related with DMI during the first GS (Table 3) and that a substantial amount of daily DMI is realized during first 60 min after the grazing event started, which is consistent with the results of studies by Enriquez-Hidalgo et al. [9] and Gregorini et al. [4].

### 4.3. Nutritive Value of the Pasture during the Grazing Down Process in the First GS

The HM influenced the DMI and the nutritive value of the residual HM during the GS. Despite the difference in HM between treatments, the nutritional value of the pasture offered was relatively similar, with only significant differences in the pre-grazing content of ADF, which is consistent with that obtained by Wims et al. [19] in pastures managed in the range of 1150 to 2000 kg DM/ha and by Pulido and Leaver [27], with ranges of HM between 1680 kg DM/ha to 2790 kg DM/ha, however the difference between MHM and HHM (+10.8 g/kg for HHM) is of relatively low biological importance, and would not influence the productive performance of animals. In several studies where significant effects of HM on the productive performance of cows were observed [8,18,27], it is feasible to observe that, when comparing high and low HM, the differences between treatments in the pre-grazing ADF content are in the range of 40 to 60 g/kg, values higher than those observed in our study. The HHM treatment was generated by delaying the regrowth period before the start of the study in relation to the regrowth period of MHM (+15 days of regrowth), which may influence fiber concentration in the pasture and, consequently, its digestibility [2,28]. However, the higher content of pre-grazing ADF and during the first 120 min of the first GS in HHM did not affect the concentration of DOMD, as fiber digestibility in a vegetative leafy spring pasture is high (>800 g/kg) and therefore an increase in ADF does not necessarily result in lower DOMD [29]. According to Chapman et al. [30], the decline in the digestibility of the pasture is more related to the progress of the growing season and, therefore, by the location of the leaves insertion points on the tiller axis during the pasture growth cycle. Given the above, it is likely that the difference in the regrowth period between treatments was not sufficient to generate changes in the DOMD.

The variation in ADF content during the first GS is directly related to changes in the morphological composition of the pasture (Table 1), due to the process of grazing and selection of cows. The highest ADF content in HHM in the first 120 m of the GS coincides with the highest proportion of pre-grazing ryegrass pseudostem that were observed in this treatment in relation to MHM, which is consistent with the results reported by Muñoz et al. [18]. Then, the ADF content was similar between treatments, which are consistent with the morphological composition at the end of the GS.

Several studies have observed that the CP content is higher in low HM pastures [31,32,33], which contrasts with that obtained in our study and Pulido and Leaver [27]. Even though there were no significant differences in the pre-grazing CP content in this experiment, it was numerically higher in MHM (+11 g/kg). Throughout the GS, selection processes were conducted by the cows and may have resulted in a higher CP concentration after the first GS for MHM compared with HHM.

However, the nutrient selection differential at the end of the first GS was similar for most of the variables, except for ADF content and a trend for DOMD. This result differs from that reported by Tharmaraj et al. [20], who observed that the pre-grazing pasture height (which caused different HMs) did not affect the selection differential. In the aforementioned study, post-grazing HM was higher in the treatment with higher pasture height (high HM), while in the present experiment post-grazing HM was similar between the treatments, as a consequence of a restricted herbage allowance (25 kg/cow/d at ground level). As a result of the above mentioned and, due to the higher pre-grazing ADF content in HHM, those cows were forced to consume a greater proportion of ADF than cows offered a MHM. On the other hand, MHM cows tended to select for DOMD.

In general, a higher intake of ADF correlates with a decrease in the availability of nutrients digested by cows, due to the greater rate of ruminal filling and decrease in the rate of digestion and passage of forage, which negatively affects milk production [34].

In the present study, the difference in ADF content was of low biological importance. However, if the highest DMI obtained in HHM is considered, it is likely to produce changes in nutrient digestion and, therefore, in the productive performance of the cows. Similarly, it should be considered that, to the extent that the following GS occur after the initial 240 min of grazing, the nutritional value of the forage in HHM should tend to be lower than in MHM, due to the progressive decrease of the amount of leaves in the pasture.

This may explain the greater productive performance of cows offered MHM compared with those offered HHM despite the greater herbage removal for cows offered HHM and that at the beginning of the GS the nutritional value was similar among HMs.

These results are relevant for strip-grazing systems, particularly when determining to allocate one or more strips per day, or to define the best moment (based on HM) at which a new grazing sector should be offered to dairy cows with the objective to combine a high pasture DMI with high nutritional value.

## 5. Conclusions

Pre-grazing herbage mass affects the structural and morphological characteristics of the pasture. The HHM pasture (~3400 kg DM/ha) used in this study caused a decrease in the bite rate during the first GS, and an increase in the bite mass and DMI in the first 60 min of the GS, compared to the MHM pasture (~2600 kg DM/ha). The grazing process was modified by the HM, affecting the DM disappearance rate of the pasture, especially during the first 60 min of the GS.

## Figures and Tables

**Table 1:** Effect of a medium (MHM) and high (HHM) pre-grazing herbage mass on sward measurements and defoliation characteristics of a pasture grazed by dairy cows.

Sward Characteristics	Treatment		SEM ^1^	*p*-Value
	MHM	HHM		
Pre-grazing herbage mass (kg DM/ha)	2662.86	3374.22	136.646	0.038
Post-grazing herbage mass (kg DM/ha)	1567.18	1677.98	43.477	0.183
Pre-grazing height (cm)	11.34	14.89	0.680	0.038
Post-grazing height (cm)	5.86	6.40	0.217	0.185
Free leaf lamina (cm)	17.58	22.79	0.376	0.002
Lamina width (mm)	4.17	5.37	0.192	0.022
Tiller density (tiller/m^2^)	9822.51	8122.09	347.873	0.041
Morphological composition (pre-grazing)				
Ryegrass leaves (g/kg DM)	690.32	451.29	41.842	<0.001
Ryegrass pseudostem (g/kg DM)	144.70	318.89	27.621	<0.001
Dicotyledonous (g/kg DM)	29.78	24.76	8.152	0.640
Dead material (g/kg DM)	135.17	202.79	30.453	0.103
Morphological composition (after 240 min of grazing)				
Ryegrass leaves (g/kg DM)	434.75	352.43	62.109	0.351
Ryegrass pseudostem (g/kg DM)	374.84	439.05	56.885	0.425
Dicotyledonous (g/kg DM)	25.29	22.56	12.214	0.856
Dead material (g/kg DM)	165.10	186.25	30.535	0.621
Defoliation characteristics				
Defoliation depth (cm)	5.48	8.48	0.653	0.053
Forage removed (kg DM/cow/day)	10.18	12.32	0.440	0.046
Area allocated per cow (m^2^/cow/day)	95.52	75.96	2.886	0.019

^1^ SEM = Standard error of the mean.

**Table 2:** Effect of medium (MHM) and high (HHM) pre-grazing herbage mass on productive performance, body condition score, and body weight of dairy cows.

Item	Treatment		SEM ^1^	*p*-Value
	MHM	HHM		
Milk production (kg/cow/day)	30.60	29.43	1.346	0.021
Milk fat concentration (g/kg)	39.80	42.87	0.495	<0.001
Milk protein concentration (g/kg)	31.29	31.61	0.451	0.637
Milk fat production (g/cow/day)	1222.03	1251.90	72.578	0.774
Milk protein production (g/cow/day)	946.88	929.38	43.889	0.780
Body condition score				
Initial	2.66	2.64	0.036	0.689
Final	2.81	2.85	0.041	0.482
Body weight (kg)				
Initial	502.45	508.96	19.849	0.818
Final	522.38	518.72	18.796	0.891

^1^ SEM = Standard error of the mean.

**Table 3:** Effect of medium (MHM) and high (HHM) pre-grazing herbage mass on grazing behavior, bite rate, bite mass, and herbage intake of dairy cows during the first grazing session.

Item	Time Interval, min	Treatment		SEM ^1^	*p*-Value ^2^
		MHM	HHM		
Grazing time (min)	0–60	59.54	59.54	0.148	1.000
	60–120	52.34	51.67	1.139	0.676
	120–180	28.64	31.52	1.180	0.085
	180–240	21.17	24.26	1.516	0.153
Ruminating time (min)	0–60	0.03	0.14	0.073	0.316
	60–120	2.98	4.75	0.691	0.071
	120–180	18.92	17.70	1.077	0.425
	180–240	24.01	21.25	1.279	0.127
Idling time (min)	0–60	0.15	0.18	0.128	0.872
	60–120	3.67	2.59	0.720	0.291
	120–180	9.72	8.50	0.886	0.333
	180–240	10.30	8.90	1.030	0.337
Bite rate (bites/min)	0	67.42	59.77	1.444	0.001
	60	65.07	54.12	1.250	<0.001
	120	60.02	52.52	1.560	0.002
	180	53.87	47.60	1.563	0.005
Bite mass (g of DM)	0–60	0.9567	1.5138	0.117	0.001
	60–120	0.2816	0.2898	0.044	0.889
	120–180	0.4975	0.3925	0.086	0.357
Herbage intake (kg DM/cow)	0–60	3.5767	4.6616	0.345	0.032
	60–120	0.9975	0.9143	0.152	0.694
	120–180	0.7135	0.6092	0.091	0.420

^1^ SEM = Standard error of the mean; ^2^ The comparison between HMs was made within each time interval (same row).

**Table 4:** Coefficients of the model used to characterize the grazing-down process and herbage disappearance rate in a pasture with a medium (MHM) and high (HHM) pre-grazing herbage mass grazed by dairy cows.

Item	Treatment		SEM ^1^	*p*-Value
	MHM	HHM		
a ^2^	2668.3	3323.0	99.17	<0.001
b ^3^	1287.0	1767.6	99.44	0.001
c ^4^	1.2565	1.4855	0.24	0.510
Herbage disappearance rate (kg DM/ha per hour)				
At time 0.5 (30 min)	630.73	1081.91	84.99	<0.001
At time 1 (60 min)	349.55	480.10	26.32	0.001
At time 2 (120 min)	153.34	144.26	20.49	0.755
At time 3 (180 min)	82.59	63.73	15.80	0.404
At time 4 (240 min)	49.28	34.47	12.01	0.388

^1^ SEM = Standard error of the mean; ^2^ a = Pre grazing herbage mass; ^3^ b = Potential herbage mass disappearance; ^4^ c = fractional disappearance rate of herbage mass (%/h).

**Table 5:** Effect of medium (MHM) and high (HHM) pre-grazing herbage mass on nutritive value of the pre grazing and residual forage during the first grazing session.

Item	Time after the Start of the GS	Treatment		SEM ^1^	*p*-Value ^2^
		MHM	HHM		
Crude protein (g/kg)	Pre-grazing	151.81	140.81	6.604	0.360
	60 min	144.16	131.62	2.339	0.064
	120 min	125.73	120.97	3.137	0.394
	180 min	126.16	117.20	1.587	0.057
	240 min	116.28	106.18	0.992	0.022
Acid detergent fiber (g/kg)	Pre-grazing	268.04	278.84	1.074	0.019
	60 min	262.72	271.43	1.123	0.032
	120 min	278.76	292.39	2.076	0.043
	180 min	288.36	297.95	2.260	0.096
	240 min	295.60	294.36	2.451	0.777
Neutral detergent fiber (g/kg)	Pre-grazing	498.69	505.71	1.495	0.080
	60 min	465.39	474.30	3.116	0.183
	120 min	476.60	491.80	1.575	0.020
	180 min	490.12	496.02	5.115	0.501
	240 min	499.03	498.99	2.797	0.994
DOMD ^3^ (g/kg)	Pre-grazing	746.78	736.36	4.228	0.224
	60 min	770.79	769.16	0.430	0.118
	120 min	754.61	739.96	2.941	0.071
	180 min	736.56	731.26	4.176	0.465
	240 min	730.29	739.43	3.347	0.223

^1^ SEM = Standard error of the mean; ^2^ The comparison between HMs was made within each time interval (same row); ^3^ DOMD = Digestible organic matter in the dry matter.

**Table 6:** Nutrient selection differential after the end of the first grazing session in a pasture with a medium (MHM) and high (HHM) pre-grazing herbage mass grazed by dairy cows.

Item	Treatment		SEM ^1^	*p*-Value
	MHM	HHM		
Crude protein	1.28	1.23	0.043	0.399
Acid detergent fiber	0.86	0.93	0.024	0.032
Neutral detergent fiber	0.97	0.99	0.026	0.538
DOMD ^2^	1.02	1.00	0.011	0.084

^1^ SEM = Standard error of the mean; ^2^ DOMD = Digestible organic matter in the dry matter.
